# Do Stretch-Shortening Cycles Really Occur in the Medial Gastrocnemius? A Detailed Bilateral Analysis of the Muscle-Tendon Interaction During Jumping

**DOI:** 10.3389/fphys.2019.01504

**Published:** 2019-12-13

**Authors:** Jeroen Aeles, Benedicte Vanwanseele

**Affiliations:** ^1^School of Human Movement and Nutrition Sciences, The University of Queensland, Brisbane, QLD, Australia; ^2^Department of Movement Sciences, KU Leuven, Leuven, Belgium

**Keywords:** jumping, architectural gear ratio, fascicle behavior, muscle-tendon unit, tendon dynamics

## Abstract

The effect of stretch-shortening cycles (SSCs) is often studied in laboratory settings, yet it remains unclear whether highly active muscle SSCs actually occur during *in vivo* movement. Nine highly trained jumping athletes performed single-leg pre-hop forward jumps at maximal effort. We hypothesized that these jumps would induce a SSC at the level of the muscle in the medial gastrocnemius. Kinematic and kinetic data were collected together with electromyography signals (EMG) and muscle fascicle length and pennation angle changes of the medial gastrocnemius of both legs and combined with a musculoskeletal model to calculate the stretch-shortening behavior of the muscle (fascicles) and tendon (series-elastic element). The length changes of the fascicles, longitudinal muscle displacement, series-elastic element, and whole muscle-tendon unit further allowed for a detailed analysis of the architectural gearing ratio between different phases of the SSC within a single movement. We found a SSC at the level of the joint, muscle-tendon unit and tendon but not at the muscle. We further found that the average architectural gearing ratio was higher during the stretching of the series-elastic element as compared to when the series-elastic element was shortening, yet this was not statistically tested because of low sample size for this parameter. However, we found no correlation when plotting the architectural gearing ratio as a function of the fascicle velocities at each instance in time. Despite the athletes having a clear preferred leg for jumping, we found no differences in any kinematic or kinetic parameter between the preferred and non-preferred leg or any parameter from the muscle-tendon interaction analysis other than a reduced longitudinal muscle shortening in the non-preferred leg (*p* = 0.008). We conclude that, although common at the level of the joints, MTUs, and tendon (series-elastic element), highly active SSCs very rarely occur in the medial gastrocnemius, even in movements that induce high loading. This has important implications for the translation of *ex vivo* findings on SSC effects, such as residual force enhancement, in this muscle. We further conclude that there is no precise tuning of the architectural gearing ratio in the medial gastrocnemius throughout the whole movement.

## Introduction

The stretch-shortening cycle (SSC) is a common phenomenon in many naturally occurring movements and has long been identified as a performance-enhancing mechanism (Bosco et al., 1982; Seiberl et al., 2015). It is defined as the rapid stretching of a pre-activated muscle prior to shortening of that same muscle (Nicol et al., 2006). This speed aspect is important because the time for muscles to produce high forces during SSCs *in vivo* is often limited (Farris et al., 2016) and would require muscles to shorten at very high velocities, impeding their capacity to produce high forces. To cope with this, the muscles rely on tendons to store elastic energy, extending the time available for active muscle contractions, allowing the muscle to shorten at lower velocities, which forms a large component of the performance enhancement from a SSC (Cavagna et al., 1965).

A SSC can be found at different levels, from the joint level muscle and tendon separately. Indeed, ever since the discovery of the decoupling of the contractile and elastic elements from the whole muscle-tendon unit (Griffiths, 1984, 1987), it has become apparent that a SSC of a muscle or tendon can occur without a SSC at the joint level. The squat jump is a typical example where a SSC is found in the tendon, but not in the muscle, muscle-tendon unit, or at the joint level (Kurokawa et al., 2001; Farris et al., 2016; Aeles et al., 2018). In a study using similar methodologies as Kurokawa et al. (2001, 2003) investigated the medial gastrocnemius muscle-tendon behavior during a counter-movement squat jump, in which one could expect that as this movement does have a SSC at the joint level, the SSC would also be found at the level of the muscle-tendon unit or muscle. The authors, however, found no differences in muscle-tendon unit, muscle, or tendon length changes in medial gastrocnemius compared to the squat jump. In a recent study, we were able to overcome the lack of MTU stretching in a classic squat jump and counter-movement jump and induced a SSC at the level of the medial gastrocnemius muscle-tendon unit by adding a specific pre-hop to the squat jump, effectively changing the behavior of the muscle and tendon of the plantar flexors (Aeles et al., 2018). This resulted in a SSC at the level of the joint, muscle-tendon unit and tendon. However, a SSC of the muscle itself was not found in any of the aforementioned studies on walking, running, and jumping. There are a few studies that did find stretching of the muscle during walking, but this generally occurred when muscle activation was low (Ishikawa et al., 2005; Chleboun et al., 2007; Maharaj et al., 2016). Two other studies have shown stretching of the muscle, both during landing tasks (Werkhausen et al., 2017; Hollville et al., 2019). However, as the main role of the muscle-tendon unit in these tasks was to dissipate energy, there was no further requirement for the muscle to do work after the landing, and a subsequent shortening was thus absent. It appears that a highly active SSC of the lower limbs is much more common at the level of the joints and in the muscle-tendon unit, and tendon and rather rare in the muscle. Because of this, our understanding of the mechanical functioning of the muscle during highly active muscle SSCs *in vivo* remains poor.

Although we lack knowledge on the direct effect of a SSC in the muscle itself, the effect of a tendon SCC on the muscle behavior and performance in humans has been investigated more often, mostly in the plantar flexors (Fukunaga et al., 2001; Lichtwark and Wilson, 2006; Farris and Sawicki, 2012; Aeles et al., 2018). Collectively, these studies have shown that the conditions for the muscle to produce force can be optimized by altering the working length and velocity of the muscle. Another mechanism that allows optimization of the working length and velocity of the muscle is the architectural gearing ratio (architectural gearing ratio) found in pennated muscles. The architectural gearing ratio allows the muscle fascicles to shorten at slower velocities than the longitudinal muscle velocity muscle (Brainerd and Azizi, 2005). Azizi et al. (2008) introduced the concept of a variable gearing mechanism in the muscle, which allows the muscle to automatically increase its architectural gearing ratio at high muscle shortening velocities. It was later found that the muscle also relies on a high architectural gearing ratio during muscle lengthening, which by allowing the fascicles to lengthen at a slower velocity than the whole muscle serves as a protective mechanism by avoiding damage from rapid fascicle lengthening (Azizi and Roberts, 2014). Much of our knowledge on the architectural gearing ratio in muscle is derived from *ex vivo* animal studies under well-controlled, single-mode conditions and the use of an architectural gearing ratio during more complex human *in vivo* movement remains mostly unexplored. Indeed, only very few studies have made attempts at investigating the use of the architectural gearing ratio in humans. Dick and Wakeling (2017) were the first to successfully calculate the architectural gearing ratio at maximal muscle shortening velocity from experimental data in humans and confirmed the variable gearing theory proposed by Azizi et al. (2008) for humans. Other studies have since calculated the architectural gearing ratio during running (Werkhausen et al., 2017) and drop landings (Hollville et al., 2019), yet all of these studies have calculated the architectural gearing ratio as a single value and using different methods. Because of this, it remains unclear whether or not the architectural gearing ratio of a muscle can be tuned within a single movement. In many human movements there are two distinct phases, a phase of long stretching and a phase of fast shortening of the tendon. Because of this, the muscle itself shortens at very different velocities and with different loads during these two phases. From the previous work done on the architectural gearing ratio, it would be expected that the architectural gearing ratio of the muscle is tuned to better fit these different conditions, however, this has never been tested.

In a recent study, we investigated if the preference for one leg over the other for jumping was associated with architectural adaptations of the muscle (Aeles et al., 2017). We found large, unexpected differences in muscle architecture between the two legs in jumping athletes and untrained individuals, but the differences were not related to leg preference for jumping. As the different architectures in this muscle may lead to different dynamic behavior during jumps, and perhaps different tuning of the architectural gearing ratio of the muscle between the two legs, we further explored this in this study.

As such, our aim for this study was to use a unilateral jump with a pre-hop, which was expected to induce an active SSC at the level of the joints, muscle-tendon unit, muscle and tendon of the medial gastrocnemius, to further explore remaining questions on the muscle and tendon behavior *in vivo*. Our first hypothesis was therefore that the single-leg forward jumping movement we chose for this study would be of high enough loading to induce an active SSC at the level of the ankle joint, medial gastrocnemius muscle-tendon unit, muscle and tendon. We also hypothesized that the architectural gearing ratio of the muscle is tuned differently between phases during which the tendon is slowly lengthening versus when it is rapidly shortening at the end of the movement. More specifically, this means that when the tendon is stretching slowly, the muscle is generally shortening at low velocities but high loads and so we hypothesize that the architectural gearing ratio will be low compared to when the muscle and tendon are shortening at high velocities. We further explored the effect of different architectures between the left and right leg on dynamic differences in the interaction between muscle and tendon.

## Materials and Methods

### Participants and Protocol

Nine highly trained jumping athletes (three female, six male, high and long jumpers; body mass: 71.1 ± 5.4 kg; body length: 182.3 ± 6.1 m) participated in this study. All participants were ranked in the top 10 in Belgium in their respective field and were injury free at least 3 months prior to testing, had no chronic lower limb injuries and were active in competition. The preferred leg for jumping for each participant was self-reported at the start of the study and was the same as the take-off leg used in competition for all participants. All participants gave written informed consent and the study was approved by the local ethics committee (Medical Ethical Committee of UZ Leuven, file number: S57477).

**FIGURE 1:**
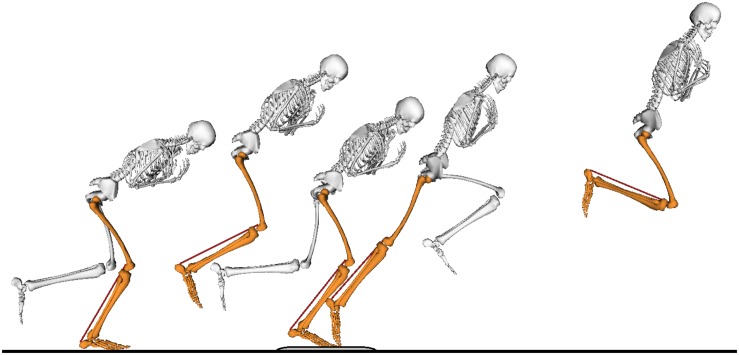
The movement task consisted of a single-leg small forward hop onto a force plate, followed by a maximal-effort forward jump. Only the jumping leg (orange) made contact with the ground during the task. The non-jumping leg (white) is shown only during ground contact for clarity of the figure.

After a 15 min warm-up consisting of running at a comfortable speed, squat jumping and countermovement jumping, the athletes performed single-leg forward jumps. To maximize the chance of inducing a SSC in the muscle-tendon unit, muscle, and tendon, the athlete started by performing a small forward hop onto the force plate on one leg, followed by a maximal-effort forward jump on the same leg (see Figure 1). The other leg did not make contact with the ground other than to stabilize the body at the start of the small forward hop and at the final landing after the maximal-effort jump. All data was analyzed for the foot-ground contact period on the force plate except for EMG data, which was analyzed throughout the whole movement to assess pre-activation. After two successful trials, the jumps were repeated for the other leg. A successful trial was defined as the foot making contact with the force plate only and clear ultrasound images. For both legs, the jump that had the greatest impulse (see below for calculations) was chosen for further analysis.

### Data Acquisition and Analysis

**FIGURE 2:**
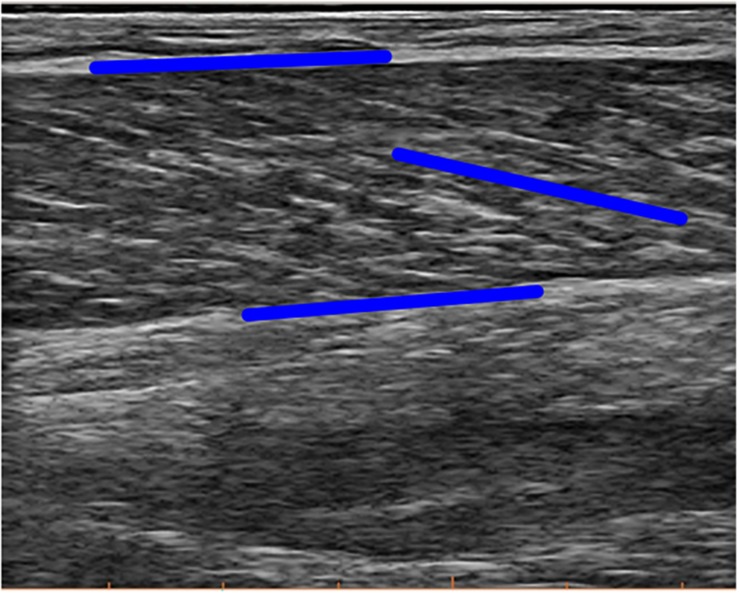
Example image of the ultrasound measurement. Blue lines illustrate tracking of the orientation of both aponeuroses and the fascicle. Note that the blue line in parallel to the fascicle here is not the final fascicle length, rather extrapolation methods were used on the local coordinates of these lines to calculate final fascicle length and pennation angle.

A linear ultrasound transducer (128 elements, 8 MHz, 60 mm field of view, UAB Telemed Lithuania) was attached to the lower leg that was used for the jumps. The transducer was placed over the mid-belly of the medial gastrocnemius muscle and aligned with the orientation of the muscle fascicles to obtain longitudinal images of the fascicles. The transducer was securely fixed on the leg with bandages and medical tape and images were sampled at 60 Hz. Ultrasound image recordings were processed by a single person as described in Aeles et al. (2018). Briefly, for each image in a recording, the superficial and deep aponeurosis and fascicle orientation were tracked using freely available software (UltraTrack, Farris and Lichtwark, 2016), see Figure 2. The reliability of the person that processed the images was reported earlier in Aeles et al. (2017) (intra-class correlations > 0.93 and standard error of measurements < 1.0 mm and 0.6° for fascicle lengths and pennation angles, respectively). Fascicle length was calculated as the extrapolated line along the fascicle orientation between the intersection points with the visible aponeuroses. This method is more robust and reliable when using the aforementioned tracking algorithm and is suitable in this muscle, which has no significant curvature of the fascicles. The muscle was also scanned along its length prior to the ultrasound transducer placement for these dynamic measurements to ensure that there was no significant curvature of either of the aponeuroses at least 2–3 cm more proximal than the location of the transducer. Pennation angles were defined as the angle between this fascicle and the deep aponeurosis. The longitudinal displacement of the muscle was calculated by multiplying the fascicle length with the cosine of the pennation angle. We further use the term longitudinal muscle shortening and longitudinal muscle lengthening when the displacement follows the direction of fascicle shortening or lengthening, respectively. The length changes of the series-elastic element (series-elastic element) were estimated as the difference in the length changes of the muscle-tendon unit and the fascicle, taking into account the pennation angle (Fukunaga et al., 2001). Fascicle and longitudinal muscle velocities were calculated by taking the time derivative of their length changes. The architectural gearing ratio of the muscle was calculated by dividing the longitudinal muscle velocity by the fascicle velocity at each data point. As to only take the AGRs during significant periods of high longitudinal muscle velocities into further analyses, we extracted the architectural gearing ratio values that corresponded to longitudinal muscle shortening at velocities faster than −25 mm/s and longitudinal muscle lengthening at velocities faster than 25 mm/s. This value was chosen based on the fact that we are confident in detecting a minimal absolute length change of 5 mm with the ultrasound technique. Combined with a minimal ground-contact time of roughly 220 ms, we rounded up the value of a minimal detectable velocity of 22.7 to 25 mm/s We then also further split these architectural gearing ratio values into phases during which the series-elastic element was lengthening (long, slow phase) and when it was shortening rapidly (at the end of the movement). Fascicle lengths are used further in the manuscript when referencing to “the muscle,” series-elastic element length changes when referencing to “the tendon.”

The ultrasound image collection was synchronized with a Vicon motion capture system for 3D motion analyses (Vicon, Oxford Metrics, United Kingdom). 42 reflective markers containing four technical clusters were placed on anatomical landmarks to make a 3D reconstruction of the motion using 10 infrared cameras sampling at 150 Hz. Ground reaction forces were collected with a force plate sampling at 1000 Hz (AMTI, United States). Impulse was chosen as performance parameter and was calculated from the 3D resultant ground reaction force by multiplying the force by time of force application. Marker locations were exported to OpenSim 3.2 (Delp et al., 2007) to scale a full-body musculoskeletal model (Hamner et al., 2010). The scaled models included a muscle-tendon unit (muscle-tendon unit) actuator representing the medial gastrocnemius. A Kalman smoothing algorithm (De Groote et al., 2008) was used to calculate joint angles from the marker coordinates, which also provided us with the joint angular velocities and the length changes of the muscle-tendon unit throughout the motion, taking into account the scaled moment arms as a function of the ankle and knee joint angles. Then, an inverse dynamics analyses was performed in the OpenSim software, using the joint angles and the ground reaction force data. Joint power was calculated from the angular velocities and joint moments and then split into negative (absorption) and positive (generation) phases. From these phases, work for each joint was calculated as the negative and positive work, respectively and net work done by the joint was calculated by subtracting the difference of the positive from the negative work. For some participants and in some joints, the movement ended with power absorption and these data were removed for the joint work calculations as they did not contribute to storage of elastic energy used during the stance phase.

EMG recordings of the medial gastrocnemius were collected from the same leg on which the ultrasound transducer was placed (ZeroWire EMG Aurion, Milano, Italy), sampling at 900 Hz. Raw EMG data signals were first band-pass filtered (20–400 Hz), rectified and then low-pass filtered (10 Hz) using a fourth-order Butterworth filter. Then, for each leg, the resulting EMG signals were normalized to the maximal activation between ground contact and toe-off during the jumps, using a moving average over five data points. To determine the timing of pre-activation before ground contact a threshold of two times the standard deviation during the relaxed standing position was used to define when the muscle was active. Due to technical issues with the EMG recordings for some participants, data from only six participants was analyzed.

### Statistical Comparisons

Comparisons between the preferred and non-preferred leg were made on the following parameters: the ground-contact time; the minimal and maximal joint angles, angular velocities, and moments, the power absorption and generation, and the negative, positive, and net work for the ankle, and knee; the peak value of the resultant GRF and the impulse, both normalized to body mass; the pre-activation of the medial gastrocnemius (*N* = 6); the amount of fascicle shortening and lengthening, pennation angles changes, the amount of longitudinal muscle displacement, and muscle-tendon unit and series-elastic element length changes; the maximal fascicle and aponeurosis displacement velocities; the average architectural gearing ratio during high longitudinal muscle shortening velocity for the whole ground contact phase (rows “All” in Table 2), and separated between the phase when the series-elastic element was lengthening and when it was shortening, and the average architectural gearing ratio during high longitudinal muscle lengthening velocity for the same phases as the shortening. Because of the small dataset (*N* = 9 unless otherwise stated), comparisons between the preferred and non-preferred leg were made using the Wilcoxon signed ranks test. Median values and interquartile ranges between the 75th and the 25th percentiles are reported. The mean values and the standard deviation are also reported for descriptive purposes but were not used for statistical testing. A linear regression analysis was performed on both datasets of architectural gearing ratio as a function of fascicle shortening or lengthening velocities and the correlation coefficients were calculated. This was done for data of the preferred and non-preferred leg combined (as their was no statistical difference in architectural gearing ratio between the two; *p* = 0.570). Alpha values were set at 0.05. Some parameters had datasets that were too small (e.g., only three participants had longitudinal muscle lengthening at velocities faster than 25 mm/s and thus the architectural gearing ratio during this lengthening was calculated for these three participants only) and were therefore not compared statistically but are discussed in a descriptive manner.

## Results

Despite the athletes having a clear preference for one leg over the other for jumping, we found no significant difference between the body mass normalized peak resultant GRF or impulse generated between the preferred and non-preferred leg (Table 1). We also found no differences between the preferred and non-preferred leg in any of the minimal or maximal joint angles, maximal angular velocities, maximal body mass normalized moments, maximal power absorption and generation, or total negative, positive or net work done at the ankle or knee joint (Table 1).

**TABLE 1:** Kinetic and kinematic variables for the preferred and non-preferred leg.

		**Preferred**	**Non-preferred**	***p*-value**
Ground contact time (s)		0.291 [0.282 0.311]	0.313 [0.281 0.328]	0.359
		(0.295 ± 0.037)	(0.307 ± 0.034)	
GRF (N/kg)	Peak	27.92 [26.83 29.36] (28.94 ± 3.76)	29.09 [25.30 31.88] (28.98 ± 3.52)	1.000
Impulse (N⋅s/kg)		0.61 [0.57 0.63]	0.63 [0.60 0.65]	0.203
		(0.61 ± 0.05)	(0.63 ± 0.03)	
**Peak joint angles (deg)**				
Ankle	DF	29 [27 32]	32 [29 34]	0.652
		(30 ± 6)	(31 ± 5)	
	PF	−30 [−31 −27]	−29, [−34 −26]	0.910
		(−29 ± 4)	(−29 ± 7)	
Knee	Flx	−58 [−61 −56]	−59 [−63 −52]	0.734
		(−58 ± 6)	(−57 ± 6)	
	Ext	−9 [−11 −5]	−5 [−11 −4]	0.734
		(−8 ± 5)	(−7 ± 3)	
**Peak joint angular velocities (deg/s)**				
Ankle	DF	397 [347 455]	360 [318 482]	0.820
		(391 ± 97)	(395 ± 113)	
	PF	−828 [−921 −777]	−845 [−995 −781]	0.820
		(−864 ± 109)	(−884 ± 141)	
Knee	Flx	−249 [−323 −169]	−232 [−310 −162]	0.570
		(−241 ± 87)	(−235 ± 81)	
	Ext	607 [575 686]	644 [590 698]	0.910
		(624 ± 97)	(617 ± 92)	
**Peak joint moments (Nm/kg)**				
Ankle	PF	−4.3 [−4.4 −3.9]	−4.3 [−4.7 −3.9]	0.821
		(−4.3 ± 0.5)	(−4.3 ± 0.6)	
Knee	Flx	−1.3 [−1.5 −1.1]	−1.1 [−1.3 −0.9]	0.164
		(−1.4 ± 0.3)	(−1.2 ± 0.6)	
	Ext	2.9 [2.6 3.2]	2.5 [2.3 2.9]	0.204
		(2.8 ± 0.5)	(2.6 ± 0.5)	
**Peak joint power (W/kg)**				
Ankle	Abs	−13.0 [−17.3 −8.1]	−12.1 [−14.5 −8.5[	0.250
		(−13.0 ± 5.1)	(−11.7 ± 3.3)	
	Gen	27.2 [26.4 34.5]	27.2 [23.3 30.2]	0.250
		(23.0 ± 5.5)	(28.4 ± 6.0)	
Knee	Abs	−4.9 [−6.0 −4.0]	−5.1 [−6.5 −4.6]	0.734
		(−5.0 ± 1.9)	(−4.9 ± 2.2)	
	Gen	10.7 [9.0 13.2]	9.2 [8.6 13.0]	0.570
		(10.8 ± 3.3)	(10.6 ± 3.1)	
**Joint work (J/kg)**				
Ankle	Neg	−1.7 [−2.0 −1.3]	−1.8 [−2.3 −1.3]	0.910
		(−1.8 ± 0.7)	(−1.9 ± 0.7)	
	Pos	4.5 [3.9 5.5]	4.3 [3.7 5.0]	0.359
		(4.8 ± 1.4)	(4.5 ± 0.9)	
	Net	3.1 [2.3 4.0]	2.4 [2.0 3.6]	0.129
		(3.0 ± 1.2)	(2.5 ± 0.7)	
Knee	Neg	−0.5 [−0.7 −0.3]	−0.6 [−0.8 −0.4]	0.570
		(−0.5 ± 0.2)	(−0.5 ± 0.3)	
	Pos	2.0 [1.5 2.6]	1.5 [1.3 2.4]	0.301
		(2.0 ± 0.7)	(1.9 ± 0.6)	
	Net	1.4 [0.9 1.9] (1.5 ± 0.7)	1.2 [0.9 1.6] (1.3 ± 0.5)	0.203

*Values are medians [25% quartile 75% quartile] (means ± SD). GRF, ground reaction force; DF, dorsiflexion, PF, plantar flexion/flexor; Flx, flexion/flexor, Ext, extension/extensor; Abs, absorption; Gen, generation; Neg, negative; Pos, positive. No significant differences between preferred and non-preferred leg were found.*

The medial gastrocnemius was activated prior to ground contact by an average of 0.82 ± 0.57 s in the preferred and by 0.63 ± 0.29 s in the non-preferred leg. There was no statistical difference between the preferred and non-preferred leg (median: 0.79 [0.70 0.91] and 0.74 [0.68 0.81], respectively; *p* = 0.313). The long pre-activation time in both legs prior to ground contact, confirms that the findings here represent active SSCs.

**FIGURE 3:**
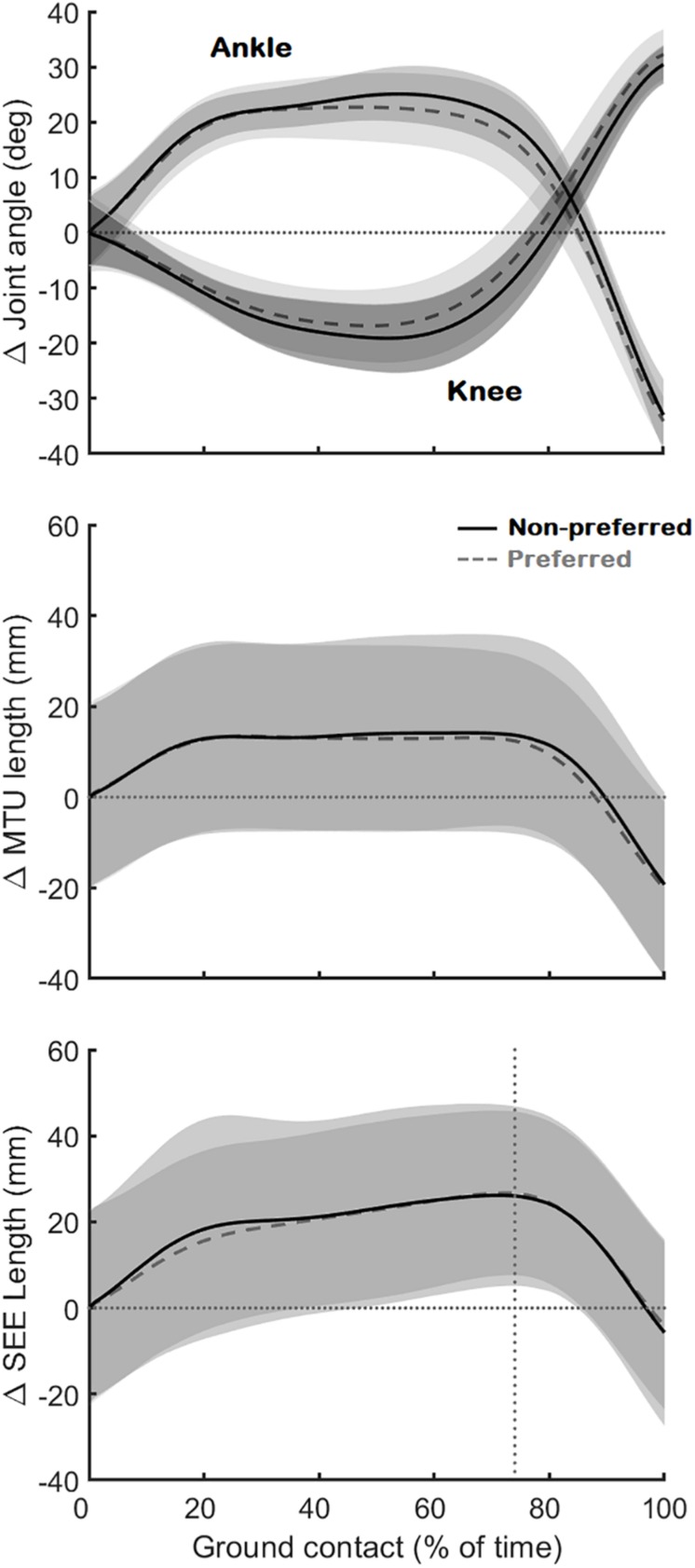
Joint angle changes of the ankle and knee **(Top)**, muscle-tendon unit (MTU) length changes **(Middle)**, and series-elastic element length (SEE) length changes **(Bottom)**. The black continuous line is the average (*N* = 10) for the preferred leg, the gray dashed line the average (*N* = 10) for the non-preferred leg. All data is shown as changes relative to the first data point at ground contact (shown as the horizontal dotted lines). The vertical dotted line on the bottom figure shows where the lengthening phase of the series-elastic element stops and the shortening phase begins.

**FIGURE 4:**
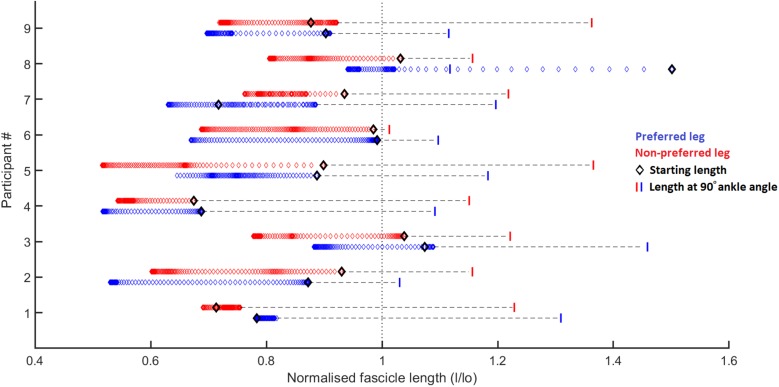
Fascicle length changes normalized to the resting fascicle length [measured at tendon slack length (data from Aeles et al., 2017)] for the preferred (blue) and non-preferred (red) leg for each participant. Each diamond represents a single data point, 1 for every 1% of the stance phase. The black diamond represents the fascicle length at the start of the ground contact phase. The vertical bars represent the fascicle length at a 90° ankle joint angle (in relaxed state, data from Aeles et al., 2017). The figure shows that most participants have little to no stretching of the fascicles following ground contact, other than participant 7 in the preferred leg. It also shows significant shortening of the fascicles in some participants (e.g., participant 2), whereas in others the fascicles behave mostly isometrically (e.g., participant 1). For all participants the fascicles at the end of the ground contact sit at (much) shorter lengths than their slack length (vertical dotted line). Considering that all participants had an ankle angle that is more dorsiflexed than 90° at initial ground contact, it is obvious that there is already considerable shortening of the fascicles (other than the preferred leg in participant 8) prior to ground contact due to pre-activation of the muscle, as shown by the horizontal dashed lines.

As expected, the movement performed by the participants results in a SSC at the level of the ankle and knee joint, medial gastrocnemius muscle-tendon unit, and series-elastic element (Figure 3). However, contrary to our first hypothesis, the muscle fascicles were stretched only notably in the preferred leg of one participant (9 mm, participant 8) and very little or not at all in all other participants (Figure 4). In some participants, the muscle fascicles shortened considerably following ground-contact whereas in others they behaved mostly isometrically.

During ground contact, the muscle fascicles shortened on average by 16 ± 9 and 14 ± 6 mm in the preferred and non-preferred leg, respectively (median: 14 [11 19] and 16 [9 18] respectively; *p* = 0.496). This coincided with a pennation angle increase of 13 ± 8° and 14 ± 9°, respectively (median: 11 [9 23] and 11 [7 17], respectively; *p* = 0.652). The fascicle shortening, combined with pennation angle changes caused a longitudinal muscle shortening of 19 ± 10 mm in the preferred leg, but only an average shortening of 14 ± 11 mm in the non-preferred leg, which was statistically lower (median: 17 [15 26] and 14 [10 18], respectively; *p* = 0.008). The muscle-tendon unit was initially stretched on average by 14 ± 5 mm in the preferred leg and 14 ± 6 mm in the non-preferred leg (median: 16 [10 18] and 15 [10 16], respectively; *p* = 1.000) (Figure 3). This was followed by a long quasi-isometric phase of the muscle-tendon unit between 20 and 80% of the ground-contact phase. Then, a rapid shortening of the muscle-tendon unit was found with average shortening amplitudes of 35 ± 3 mm in the preferred leg and 35 ± 6 mm in the non-preferred leg (median: 34 [33 37] and 35 [30 39] respectively; *p* = 0.910). The series-elastic element stretched on average by 29 ± 9 mm in the preferred leg and 27 ± 4 mm in the non-preferred leg (median: 30 [23 33] and 25 [24 30] respectively; *p* = 0.820). The stretching phase took up the majority of the ground-contact for the series-elastic element (as shown by the vertical bar in Figure 3), with rapid shortening by 34 ± 4 and 33 ± 8 mm in the preferred and non-preferred leg, respectively near the end of the movement (median: 34 [31 36] and 34 [28 38], respectively; *p* = 1.000).

The maximal shortening velocity of the fascicles was not statistically different between the preferred leg and non-preferred leg with values of 211 ± 186 and 191 ± 123 mm/s, respectively (median: 162 [137 204] and 181 [92 225], respectively; *p* = 1.000). The maximal longitudinal muscle shortening velocity was 248 ± 206 compared to 168 ± 68 mm/s for the preferred leg and non-preferred leg, respectively. Despite the large differences, this did not result in a statistically significant difference between the two legs (median: 181 [149 261] and 157 [111 235], respectively; *p* = 0.652). However, this did result in a statistically significant difference between the maximal fascicle shortening velocity and maximal longitudinal muscle shortening velocity in the preferred leg (*p* = 0.012), but not in the non-preferred leg (*p* = 0.570). Maximal stretching velocity of the muscle-tendon unit was 285 ± 106 mm/s in the preferred leg and 277 ± 115 mm/s in the non-preferred leg (median: 337 [202 365] and 270 [214 332], respectively; *p* = 0.820). Maximal shortening velocity of the muscle-tendon unit was 600 ± 74 mm/s in the preferred leg and 617 ± 110 mm/s in the non-preferred leg (median: 593 [524 667] and 589 [545 715], respectively; *p* = 0.820). The maximal stretching velocity of the series-elastic element was 383 ± 230 mm/s in the preferred leg and 383 ± 122 mm/s in the non-preferred leg (median: 341 [276 409] and 364 [285 487], respectively; *p* = 0.570). Maximal shortening velocity of the series-elastic element was 607 ± 109 mm/s in the preferred leg and 611 ± 96 mm/s in the non-preferred leg (median: 596 [525 688] and 592 [543 714], respectively; *p* = 1.000). No statistical differences were found between the preferred and non-preferred leg in any of these parameters. The maximal shortening velocities of the fascicles and the longitudinal muscle were statistically lower than the maximal shortening velocities of the muscle-tendon unit and series-elastic element in both the preferred (*p* = 0.008 for both) and the non-preferred leg (*p* = 0.004 for both).

**FIGURE 5:**
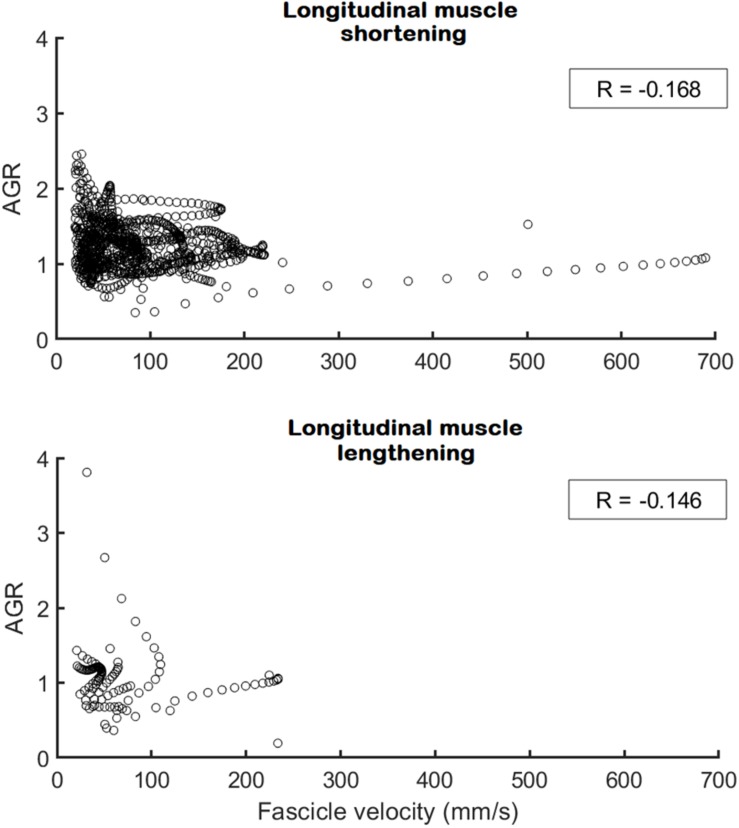
Architectural gearing ratio as a function of fascicle velocity for all participants during longitudinal muscle shortening at velocities > 25 mm/s (*N* = 9; **Top**) and during longitudinal muscle lengthening at velocities > 25 mm/s (*N* = 3; **Bottom**). Each circle represents a single data point for both preferred and non-preferred leg data combined. A very low, negative correlation coefficient (in box) was obtained for both datasets. Note that the *x*-axis on the top panel is fascicle shortening velocity whereas on the bottom panel this is fascicle lengthening velocity.

No differences between the preferred and non-preferred leg were found for the architectural gearing ratio analysis (Table 2). During series-elastic element stretching and shortening, respectively, we found phases of longitudinal muscle shortening velocities greater than 25 mm/s in the preferred leg for eight and seven athletes and in the non-preferred leg for nine and eight athletes. In order to compare values only for athletes that had longitudinal muscle shortening velocities greater than 25 mm/s in both phases, the descriptive comparisons were made for five athletes (Table 2, gray panel). There appears to be a trend in which the architectural gearing ratio seems to be greater during series-elastic element lengthening than during series-elastic element shortening. Only three participants had a period of longitudinal muscle lengthening at velocities greater than 25 mm/s, hence the results of only these three participants are shown in Table 2 (bottom, white panel). Because of the low number of participants for this parameter, the results of these comparisons are presented but not further discussed. This could be a point of interest for future studies. The correlation coefficients for the architectural gearing ratio as a function of fascicle shortening velocity during both longitudinal muscle shortening and lengthening at velocities greater than 25 mm/s were very low (Figure 5).

**TABLE 2:** Architectural gearing ratios during various phases of ground contact for the preferred and non-preferred leg.

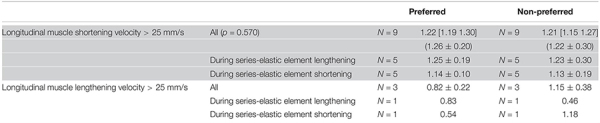

*Values are medians [25% quartile 75% quartile] (means ± SD) for the top row on which a statistical analysis was performed and means ± SD for all other rows. An architectural gearing ratio > 1 means faster longitudinal muscle velocity than fascicle velocity. To make a fair comparison, for parameters with *N* lower than 9, only the participants that could be compared between preferred and non-preferred and between series-elastic element shortening and series-elastic element lengthening phases were included, resulting in *N* = 5 for phases of high longitudinal muscle shortening velocities (gray panel). The *p*-values in the gray panel are for the comparison between preferred and non-preferred leg. Only three athletes had phases during which the aponeurosis was lengthening at high velocities (white panel). Of these three athletes only one athlete had high longitudinal muscle lengthening velocities during both the series-elastic element lengthening and series-elastic element shortening.*

## Discussion

In this study, we aimed to explore in detail the muscle-tendon interaction in the medial gastrocnemius during a movement involving a SSC. Contrary to our hypothesis, the SSC did not generally occur in the muscle fascicles, but it did result in a SSC at the level of the joints (i.e., a rapid countermovement), medial gastrocnemius muscle-tendon unit and tendon (measured as series-elastic element). We further investigated whether in a single movement the architectural gearing ratio of a muscle is tuned differently in the phases during which the series-elastic element is stretching versus shortening. We found that when the series-elastic element is stretching, the average architectural gearing ratio is slightly higher than when it is shortening, but interestingly, at instantaneous time points throughout the movement this was not related to the fascicle shortening velocity. We are also the first to compare the muscle-tendon interaction during a dynamic movement between the preferred leg used for jumping and the non-preferred leg. Despite the athletes having a clear preference on which leg to use for jumping, we did not find differences in any kinematic or kinetic parameter, activation of the muscle, or muscle-tendon interaction other than a reduced longitudinal muscle shortening in the non-preferred leg compared to the preferred leg.

### Absence of a SSC in the Muscles Fascicles

Our results show that even during high-loading movement tasks, a highly active SSC of the medial gastrocnemius muscle in humans is rare, which is not in agreement with our initial hypothesis (Figure 4). This is consistent with previous studies on the medial gastrocnemius muscle during walking, running, and bilateral jumping (Fukunaga et al., 2001; Lichtwark and Wilson, 2006; Farris and Sawicki, 2012; Aeles et al., 2018) and with results from other triceps surae muscles such as the lateral gastrocnemius (Farris et al., 2016) and soleus (Cronin and Finni, 2013; Lai et al., 2015). In a recent study on the muscle-tendon interaction in the vastus lateralis muscle, also no active stretching of the muscle fascicles was found during a squat jump (Nikolaidou et al., 2017). In the study by Nikolaidou et al. (2017), the fascicles did lengthen in the counter-movement jump, however, this occurs prior to activation of the muscle, as can be seen from the EMG data, and is therefore not considered an active SSC. In other studies that have shown stretching of the muscle fascicles in the medial gastrocnemius and soleus, vastus laterlias, and tibialis posterior, there is a similar trend of very low activation during fascicle stretching (Ishikawa et al., 2005; Chleboun et al., 2007; Maharaj et al., 2016, respectively). This suggests that muscle fascicles do sometimes stretch but never at high activation levels. The contradictory findings in studies in the same muscles (medial gastrocnemius and soleus) and during the same task (walking) warrant further investigation. The loading induced during the ground contact phase in the current study was high as the ankle and knee joint moments and positive work in the ankle were, respectively about 1.8, 1.2, and 3 times higher compared to the bilateral pre-hop squat jump in our previous study (Aeles et al., 2018). However, this did not result in a stretching of the muscle fascicles, other than in the preferred leg of only one participant. We did not find a clear explanation in any of the other data as to why this participant showed lengthening in this one leg, but this could be a noteworthy focus of a future study. The loading induced by this movement was far greater than in the step-down task in the study by Werkhausen et al. (2017) (peak ankle moment of 4.3 Nm/kg in our study versus 1.8 Nm/kg in Werkhausen et al., 2017), who did find an average stretching of about 20 mm for the medial gastrocnemius fascicles, suggesting that the stretching of muscle fascicles is not fully dependent on the loading. In two other studies looking at similar tasks, similar fascicle stretching (23 and 16 mm) was found (Spanjaard et al., 2007 and Hollville et al., 2019 respectively). This striking difference in muscle-tendon interaction between both the step-down and drop landing tasks and our single-leg jumping movement can be explained by the wide variety of roles played by the elastic tissues in muscle-tendon systems (Roberts and Azizi, 2011). Because the single-leg jump was followed by a maximal forward jump, the potential energy from the initial pre-hop is better stored and recycled later on to power the maximal jump, as dissipating this energy would be wasteful.

### Energy Dissipation Versus Energy Recycling

These different roles for the muscle-tendon system are carefully regulated by the nervous system by adapting the timing and magnitude of muscle activation. Comparing the activation of the muscle between the different studies shows that during the step-down and drop landing tasks, the medial gastrocnemius has peak activation at the start of the ground contact and then rapidly declines (Spanjaard et al., 2007; Werkhausen et al., 2017; Hollville et al., 2019). This allows for the muscle fascicles to act quasi-isometrically or even shorten slightly during the initial loading, when the forces rise rapidly, before finally lengthening during the phase of force decay. This initial active shortening prior to the passive lengthening of the muscle fascicles has previously been suggested to attenuate the high lengthening velocities of the muscle-tendon unit to much lower velocities in the muscle fascicles, serving as a protective buffer and reducing muscle damage (Proske and Morgan, 2001; Lieber and Fridén, 2002; Konow et al., 2012). In the single-leg jump, however, the medial gastrocnemius muscle stays active for a much longer period, only decreasing its activation around 80% of the ground-contact phase, allowing for a long period of stretching of the series-elastic element, storing significant amounts of potential energy to be used later in the movement, when both the series-elastic element and muscle-tendon unit are recoiling at high velocities. Combined, our findings suggest that highly active SSCs of the medial gastrocnemius muscle are unlikely to occur in human movement when the main role for the elastic tissues is not to dissipate energy. This may especially be the case in MTUs that have considerable in-series compliance, such as the triceps surae muscles. It is also noteworthy to take into account that the participants in this study were all highly trained jumping athletes. Considering the high forces that are likely acting on the lower limb muscles, it is possible that lesser trained individuals may not be able to avoid fascicle lengthening. This could be an interesting focus point for future studies.

### The Effect on the Tendon and Muscle-Tendon Unit

Considering the high loading, it could be expected that the muscle-tendon unit of the medial gastrocnemius would stretch more as compared to other movements, resulting in greater stretching of the series-elastic element and a greater amount of energy storage. However, it is likely that the peak ankle dorsiflexion angle during ground contact is close to the maximal range of motion for this joint, limiting further stretching of the muscle-tendon unit. Extending the knee more would allow for further stretching in the muscle-tendon unit of the medial gastrocnemius, but would come at the cost of less stretching of the MTUs of the knee extensors, which have also been shown to store and release significant amounts of elastic energy in the series-elastic element (Nikolaidou et al., 2017). On the other hand, at ground contact, the ankle was already in a more dorsiflexed position compared to the bilateral pre-hop squat jump while the initial knee joint angle is very similar (Aeles et al., 2018). This would mean that the muscle-tendon unit already starts at a longer length in the single-leg jump upon initial ground contact, potentially causing more total elastic energy to be stored in it. Furthermore, the fact that the muscle fascicles are already at a shorter length at the start of ground contact compared to their length at a similar joint angle in rest (Figure 4) suggests that there must already be energy being stored in the series-elastic element. This could explain why both the muscle-tendon unit and series-elastic element also have much higher maximal shortening velocities in the single-leg compared to the bilateral jump (1.3 and 1.6 times for muscle-tendon unit and series-elastic element, respectively). In our study on the pre-hop squat jump we were surprised to find that the net work in the ankle was reduced considerably in the pre-hop squat jump compared to a standard squat jump and we argued that the additional negative work that can be recycled in the former but not in the latter should have allowed for more positive work to be done in the ankle during the pre-hop squat jump (Aeles et al., 2018). We suggested that further increasing the positive work done in the ankle may interfere with the correct execution of the jump, as the joint moments need to be balanced (Farris et al., 2016). This limitation may not be applicable to the single-leg forward jump used in the current study, potentially explaining the ability of the ankle to stretch the medial gastrocnemius series-elastic element more and indeed produce much greater positive work and in result have much more and faster recoil shortening of the series-elastic element when the stored energy is released.

### The Fine-Tuning of the Architectural Gearing Ratio

The decoupling of the muscle fascicles from the entire muscle-tendon unit allowed the medial gastrocnemius fascicles to shorten at much lower maximal velocities compared to the muscle-tendon unit and series-elastic element. This is similar to what was previously shown in other tasks such as walking, running, and bilateral jumping (Fukunaga et al., 2001; Kurokawa et al., 2001, 2003; Lichtwark and Wilson, 2006; Farris and Sawicki, 2012; Aeles et al., 2018). Despite this, the muscles fascicles still shorten at high velocities compared to walking and running (Lichtwark and Wilson, 2006), yet still at less than one third of their expected maximal shortening velocities (Geyer and Herr, 2010). Our results show that the single-leg jump also has the two distinct phases found in the medial gastrocnemius muscle-tendon interaction in other movements, namely an initial phase of long stretching of the series-elastic element, which usually takes up between 60 and 85% of the ground contact phase, and a rapid shortening of the series-elastic element at the end of the movement. Contrary to our hypothesis, we found a trend for the architectural gearing ratio to be higher during the stretching of the series-elastic element, compared to when the series-elastic element is shortening. However, we noticed that the peak shortening velocity of the fascicles occurred during the stretching phase of the series-elastic element in many of our participants rather than in the shortening phase of the series-elastic element as we expected. Combined, these findings agree with the theory that the muscle is able to tune its architectural gearing ratio based on the demands of the task. This is also in agreement with an earlier study on the architectural gearing ratio measured in humans during cycling (Dick and Wakeling, 2017). However, all aforementioned studies have only reported the architectural gearing ratio during a single instance in time. Yet, if the architectural gearing ratio of a muscle would actually be tuned to the requirements of the task, one would expect the architectural gearing ratio to be relatively constant during a specific task or at least to be highly correlated with the instantaneous velocity of the fascicles. When plotting the architectural gearing ratio as a function of the fascicle shortening velocity at instantaneous time points throughout the whole movement, however, very low correlation coefficients were found when the fascicles were shortening as well as when they were lengthening (Figure 5). This finding is in contrast with our expectation that the architectural gearing ratio of the muscle is tuned precisely throughout the movement to accommodate the requirements of the task. Nonetheless, when the fascicles are shortening, the medial gastrocnemius muscle still mostly works at an architectural gearing ratio greater than 1, resulting in greater longitudinal muscle velocities relative to the fascicle velocities. In the preferred leg, this resulted in significantly greater longitudinal muscle shortening velocities relative to the muscle fascicles. The fascicle lengthening velocities reported here are also much lower compared to the values found during dissipation tasks as reported by Werkhausen et al. (2017) and Hollville et al. (2019).

### Concluding Remarks on Lack of Bilateral Differences and Limitations

This study was the first to make a bilateral comparison on the muscle-tendon interaction during a dynamic movement. We found no differences between the preferred and non-preferred leg in most of the tested parameters, despite the athletes having a clear preference for which leg to use for jumping. In an earlier study we found high variability in the medial gastrocnemius muscle architecture bilaterally (Aeles et al., 2017). Visual inspection of the fascicle length changes during ground contact from the single-leg jumps (Figure 3) also suggests that they behave very similarly in both legs, even regardless of preference. This is interesting because when two fascicles have different resting lengths, but are shortened a similar amount, assuming similar sarcomere lengths, the shorter fascicle would have greater shortening per sarcomere. This would then further result in greater shortening velocities per sarcomere, which would lower the maximal capacity of the sarcomeres to produce force. In theory, this would cause an imbalance in the force generating capacity between both muscles. This imbalance, however, could be compensated for by various mechanisms such as differences in neural drive to the muscle, or other agonistic muscles.

The small sample size in this study is a potential limitation which may affect the interpretation of the statistical findings presented here. However, because of the fairly one-sided findings in both the comparison between preferred and non-preferred leg and in the lack of muscle fascicle stretching in most participants, we suspect this sample represents the specific population. Because of the low numbers we were able to use for the architectural gearing ratio analysis, we would encourage others to perform this analysis on a larger dataset.

## Conclusion

Although the SSC of muscle is a phenomenon worthy of study, we argue that it only rarely occurs at high activation levels during *in vivo* human movement, at least in muscles that have high in-series compliance such as the medial gastrocnemius. The rare occurrence of highly active SSCs should be considered when discussing the potential effects of mechanisms such as residual force enhancement *in vivo*. Whether or not muscles actively lengthen during high loading movements is not just of relevance for performance but also in terms of injury. We further conclude that the architectural gearing ratio of the medial gastrocnemius muscle is not precisely tuned and there is high variability between data points within a single movement, and that the medial gastrocnemius muscle-tendon interaction between a preferred and non-preferred leg for jumping is very similar.

## Data Availability Statement

The datasets generated for this study are available on request to the corresponding author.

## Ethics Statement

The studies involving human participants were reviewed and approved by the Ethics Committee Research UZ/KU Leuven. The patients/participants provided their written informed consent to participate in this study.

## Author Contributions

JA was involved in project design, participant recruitment, data analyses, figure creation, and manuscript writing. BV was involved in project design and manuscript writing.

## Conflict of Interest

The authors declare that the research was conducted in the absence of any commercial or financial relationships that could be construed as a potential conflict of interest.
